# Investigating the microbiota of fermented fish products (*Pla-ra*) from different communities of northeastern Thailand

**DOI:** 10.1371/journal.pone.0245227

**Published:** 2021-01-14

**Authors:** Rutchanee Rodpai, Oranuch Sanpool, Tongjit Thanchomnang, Arporn Wangwiwatsin, Lakkhana Sadaow, Weeraya Phupiewkham, Patcharaporn Boonroumkaew, Pewpan M. Intapan, Wanchai Maleewong

**Affiliations:** 1 Department of Parasitology, Faculty of Medicine, Khon Kaen University, Khon Kaen, Thailand; 2 Mekong Health Science Research Institute, Faculty of Medicine, Khon Kaen, Thailand; 3 Faculty of Medicine, Mahasarakram University, Mahasarakram, Thailand; 4 Department of Biology, Faculty of Science, Khon Kaen University, Khon Kaen, Thailand; University of Maine, UNITED STATES

## Abstract

DNA-sequencing was performed on the V3-V4 regions of 16S rRNA genes to investigate the microbial diversity of five samples of fermented freshwater fish (*pla-ra*) from three provinces in northeastern Thailand. The samples had salt concentrations ranging from 7 to 10%, pH values from 4.83 to 7.15, and D-/L-lactic acid concentrations of 90 to 450 mg/l. A total of 598 operational taxonomic units were annotated at various taxonomic ranks based on the SILVA Database. The lactic-acid and halophilic genera *Tetragenococcus*, *Halanaerobium* and *Lactobacillus* were among the dominant taxa of bacteria. The top 20 non-redundant taxa were considered in more detail. In two *pla-ra* samples, *Tetragenococcus muriaticus* was commonly identified. *Halanaerobium fermentans* was the most abundant species in a third sample and co-dominant in another sample. *Lactobacillus rennini* was dominant in the *pla-ra* sample from Roi Et Province. Additionally, other beneficial bacteria were detected including *Staphylococcus nepalensis*, *Lactobacillus sakei*, *Lactobacillus pentosus*, *Weissella confusa*, and *Bifidobacterium bifidum*. Differences between samples may be due to use of different raw materials, salt concentrations, recipes, processes and fermentation periods. The microbial communities in *pla-ra* provide a better understanding of the production outcomes of traditional products. Further optimization of the fermentation process, for example by using dominant bacterial taxa in starter cultures, may improve processes of food fermentation, food quality and flavor control, providing useful guidelines for industrial applications.

## Introduction

Fermented fish, known as *pla-ra* or *pla-daek*, is a traditional product used as a condiment and a main dish by people in Thailand and Lao PDR [1]. *Pla-ra* has been consumed daily by Thai people since ancient times [2]. It is a famous ingredient used as seasoning in the north-eastern (*Issan*) food of Thailand, such as “*namprik pla-ra”*, “*kang lao”*, “*namya”*, and “*somtum pla-ra”* (a green papaya salad) which is popular in every region of Thailand [3, 4]. *Pla-ra* is in very high demand, with 20,000–40,000 tons/year being produced for both domestic use and export (worth about 800 million baht/year) [5]. Given this, the National Bureau of Agricultural Commodity and Food Standards has set quality standards for *pla-ra* [6] and suggested that *pla-ra* has an impact on the national economy.

*Pla-ra* is produced by fermenting raw fish with rice bran or roasted rice flour and salt in a closed container for at least six months to a year. *Pla-ra* has a very strong smell, and its salty and sour flavors depend on the amount of salt added and of lactic acid resulting from the fermentation process. The fermented products are usually stored at ambient temperatures and can last for a year or longer in the tropical climates of Southeast Asia. It is considered that longer fermentation makes the product taste better [3]. Rice bran serves as a source of carbon for fermenting microorganisms [7]. The most abundant amino-acid in *pla-ra* is glutamic acid, which suggests that the preferred taste in these fermented fish products is related to umami substances [7, 8]. *Pla-ra* varies greatly in flavor from community to community and between regions, and customers often have strong preferences for particular sources [2, 4]. In the northeastern part of Thailand, *pla-ra* is usually produced at home for family consumption, with excess being sold at local markets [9].

The quality and flavor of *pla-ra* may be related to its microbial community [10]. For example, to improve the flavor of fermented products, a microbial starter containing species such as *Tetragenococcus halophilus* or *Staphylococcus* sp. SK1-1-5 has been used in fish sauce production [10–12]. Variation in the microbial community may lead to differences in the quality, nutritional value and health benefits of fermented products [9, 13]. However, no bacterial starter cultures use in *pla-ra* processing. Therefore, knowledge of the microbial diversity in *pla-ra* is important. Although several studies have investigated communities of microorganisms in fermented fish products including *pla-ra*, there is little information from studies that did not depend on culturing microorganisms for identification [1, 5]. Using culture-independent techniques and sequencing of 16S rRNA, various lactic acid bacteria and/or halophilic or salt-tolerant bacteria have been identified from *pla-ra* products [1, 5]. However, the microbial diversity specifically found in *pla-ra* samples from northeastern Thailand is still unknown. In this work, we have identified the native bacterial communities in conventional fermented *pla-ra* from different areas of northeastern Thailand based on analysis of DNA sequences from the V3-V4 region of 16S rRNA gene. Sequences were generated using next-generation sequencing technology without the need for isolation and cultivation of the microorganisms. The results should improve our understanding of the differences in bacterial diversity between northeastern communities, thereby helping us to select appropriate functional bacteria for manufacturing high-quality and distinctive *pla-ra* products.

## Materials and methods

### Fermented-fish samples

Samples of fermented fish (*pla-ra*) were obtained from markets in three provinces in northeastern Thailand; Khon Kaen (3 markets), Kalasin (1 market) and Roi Et (1 market). Information about the products was provided by the sellers. In each case, the three main ingredients were raw freshwater fish, salt, and rice bran for fermentation (Table 1). The samples were collected between January 1st and February 5th, 2019. After purchase, the samples were place in foam boxes for transport to the laboratory. Aliquots taken for DNA preparation were frozen at -20°C to minimize further microbial activity until use. In triplicate, a ten-fold dilution of each sample using deionized water was prepared to investigate salt concentration using a salt meter (DRETEC Co., LTD, Saitama, Japan). The pH of each *pla-ra* sample was directly measured with a pH meter (Mettler Toledo, Columbus, OH). Lactic acid concentrations were measured using a QuantiQuik^TM^ D-/L-lactic acid test strip (BioAssay Systems, Hayward, CA) following the manufacturer's instructions. These measurements were performed on the day when the sample was first thawed.

**Table 1:** Production region, salinity, pH, and D-/L-lactic acid content of products used in this study. In each case, a mixture of fish was used, and fermentation lasted >1 year.

Sample^a^ name	Location (latitude and longitude)	Salinity^b^ (% w/v)	pH ± sd	D-lactic acid (mg/l)	L-lactic acid (mg/l)	Total lactic acid (mg/l)
KK1	Nonmuang, Khon Kaen (16.489 N 102.813 E)	9	5.13 ± 0.02	225.0	225.0	450.0
KK2	Nong Ruea, Khon Kaen (16.493 N 102.433 E)	10	5.02 ± 0.01	112.5	225.0	337.5
KK3	Ban Thum, Khon Kaen (16.442 N 102.722 E)	7	7.15 ± 0.04	45.0	45.0	90.0
RE	Nai Mueang, Roi Et (16.051 N 103.649 E)	7	5.15 ± 0.02	112.5	225.0	337.5
KS	Yang Talat, Kalasin (16.400 N 103.371 E)	9	4.83 ± 0.03	225.0	225.0	450.0

^a^ one sample each.

^b^ In each case, all three readings were identical.

### Bacterial DNA extraction and sequencing

Total DNA was isolated directly from the *pla-ra* samples (200 mg per sample) using a QIAamp® DNA stool mini kit (Qiagen, Hilden, Germany) as recommended by the manufacturers. Briefly, the *pla-ra* samples were extracted in optimized buffers in combination with inhibitEX buffer (Qiagen) to remove PCR inhibitors. Proteinase K was subsequently added. DNA from lysates was isolated using a DNA-binding silica-gel membrane column. Remaining impurities were efficiently removed in two wash steps. Amplification-ready DNA was then eluted in low-salt buffer (Qiagen). DNA concentration and purity were monitored using a NanoDrop™ One Spectrophotometer (Thermo Fisher Scientific, Waltham, MA) and electrophoresis through 1% agarose gels. Each DNA sample was then diluted to 1 ng/μl using sterile water.

The V3-V4 regions of the 16S rRNA gene were amplified using universal region-specific primers 341F (5’-CCT AYG GGR BGC ASC AG-3’) and 806R (5’-GGA CTA CNN GGG TAT CTA AT-3’) tagged with sample-identifying barcodes. All PCR reactions were carried out with Phusion® High-Fidelity PCR Master Mix (New England Biolabs, Ipswich, MA). The PCR cycling conditions were as follows: initial denaturation at 98°C for 1 min, followed by 30 cycles at 98°C for 10 s, 50°C for 30 s, and 72°C for 30 s, and a final extension step at 72°C for 5 min. The PCR products (400–450 bp in length) were quantified, qualified and used for library preparation. The libraries were generated using an Ion Plus Fragment Library Kit (Thermo Fisher Scientific) and quantified using Qubit and qPCR, then sequenced on an IonS5^TM^XL instrument (Thermo Fisher Scientific). Single-end reads were generated.

### Analysis and annotation of operational taxonomic units (OTUs)

Single-end reads were assigned to samples based on their unique barcode and truncated by cutting off the barcode and primer sequence. Quality filtering on the raw reads was carried out under specific filtering conditions in order to obtain the high-quality clean reads [14] using Qiime (V1.7.0, http://qiime.org/scripts/split_libraries_fastq.html) [15]. The reads were compared with the reference database (Gold database) using the UCHIME algorithm (http://www.drive5.com/usearch/manual/uchime_algo.html) [16] to detect chimeric sequences. Any such sequences were removed using the UCHIME algorithm [17]. From the effective reads (Table 2), sequences sharing ≥ 97% similarity were assigned to the same OTU using Uparse software (Uparse v7.0.1001) [18]. A representative sequence from each OTU was screened for further annotation. Mothur software was used to compare our sequences against the SSU rRNA database, SILVA (http://www.arb-silva.de/), for species annotation at each taxonomic rank [19].

**Table 2:** Quality-control information, sequence length-distribution and OTU clustering.

Sample	Raw reads	Clean reads	Base (nt)	Avg. Length (nt)	Q20	Effective (%)	OTUs	Unique OTUs
KK1	112427	90158	38683375	429	90.59	80.19	200	30
KK2	95143	90373	38612344	427	89.13	94.99	354	116
KK3	93567	90319	38764167	429	87.82	96.53	204	29
RE	78982	75132	32301400	429	82.68	95.13	235	81
KS	100129	90111	38130981	423	86.47	89.99	239	46

Raw reads means reads after filtration; Clean reads means reads after removal of chimeras; Base means base number of nucleotides in clean reads; Avg. Length means average length of clean reads; Q20 means the percentage of bases of quality greater than 20; Effective **(%)** means the percentage of clean reads relative to raw reads; OTUs means the number of OTUs identified in different samples; Unique OTUs means the number of OTUs only identified in a single sample.

### Accession numbers

All sequence reads have been deposited at the NCBI Sequence Read Archive (SRA) under project accession number PRJNA657478.

## Results

### Physicochemical properties of *pla-ra*

Details of salinity, pH, and D-/L-lactic acid content of each *pla-ra* sample are presented in Table 1. Salt content ranged from 7% to 10%, while the pH value range was 4.83–7.15. The highest pH level (7.15) was in the sample from Ban Thum market in Khon Kaen (KK3). The highest level of total lactic acid was 450 mg/l in the samples from Nonmuang market in Khon Kaen (KK1) and Yang Talat, Kalasin (KS). In samples from Nong Ruea market of Khon Kaen (KK2) and Nai Mueang, Roi Et (RE), total lactic acid was approximately 337.5 mg/l with L-lactic acid level being higher than D-lactic acid level. The sample from KK3 contained the lowest total lactic acid level, at approximately 90 mg/l.

### Microbial diversity in *pla-ra*

**Fig 1:**
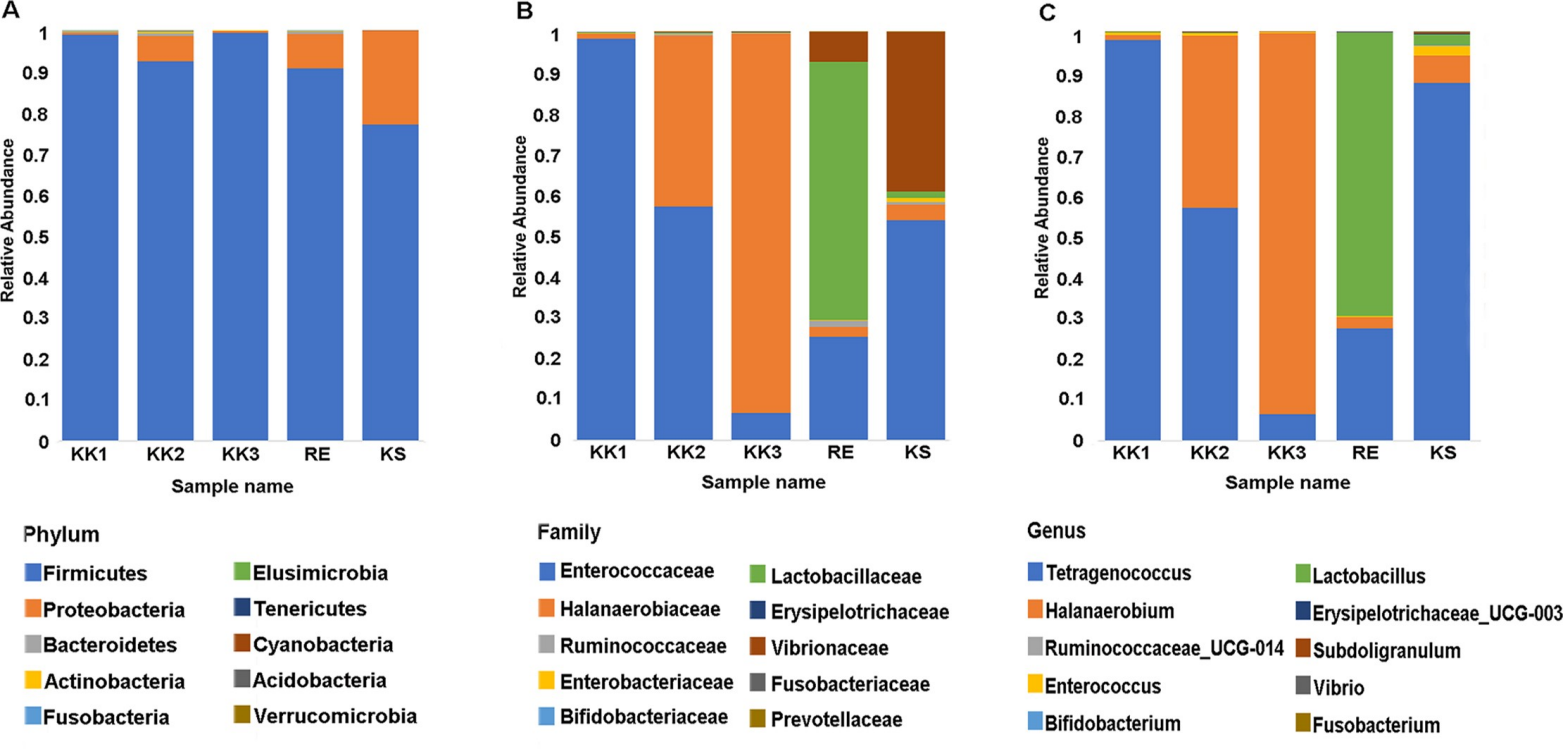
The top 10 taxa in terms of relative abundance at each taxonomic rank. A) Phylum, B) Family, C) Genus.

Amplicons from the V3-V4 regions of 16S rRNA gene were sequenced to generate raw reads, and chimeric reads were filtered out. The output statistics are shown in Table 2. Sequences sharing ≥ 97% similarity were assigned to the same OTU. A total of 598 OTUs in 17 phyla were identified from the five *pla-ra* samples (S1 Table). The top 10 phyla (in terms of numbers of sequences) were considered in more detail. Firmicutes (77.1–99.4%) was the most-represented phylum in all samples (Fig 1A), while the top 10 families and genera varied among samples (Fig 1B and 1C). For the sample KK1, reads from the genus *Tetragenococcus* (Enterococcaceae), were by far the most abundant. Families Enterococcaceae and Halanaerobiaceae (genera *Tetragenococcus* and *Halanaerobium*, respectively) were abundant in sample KK2. In sample KK3, *Halanaerobium* (Halanaerobiaceae) was predominant. The families Lactobacillaceae and Enterococcaceae were abundant in sample RE, with smaller contributions from Vibrionaceae and Halanaerobiaceae. Families Enterococcaceae (genus *Tetragenococcus*) and Vibrionaceae were most abundant in the KS sample.

**Fig 2:**
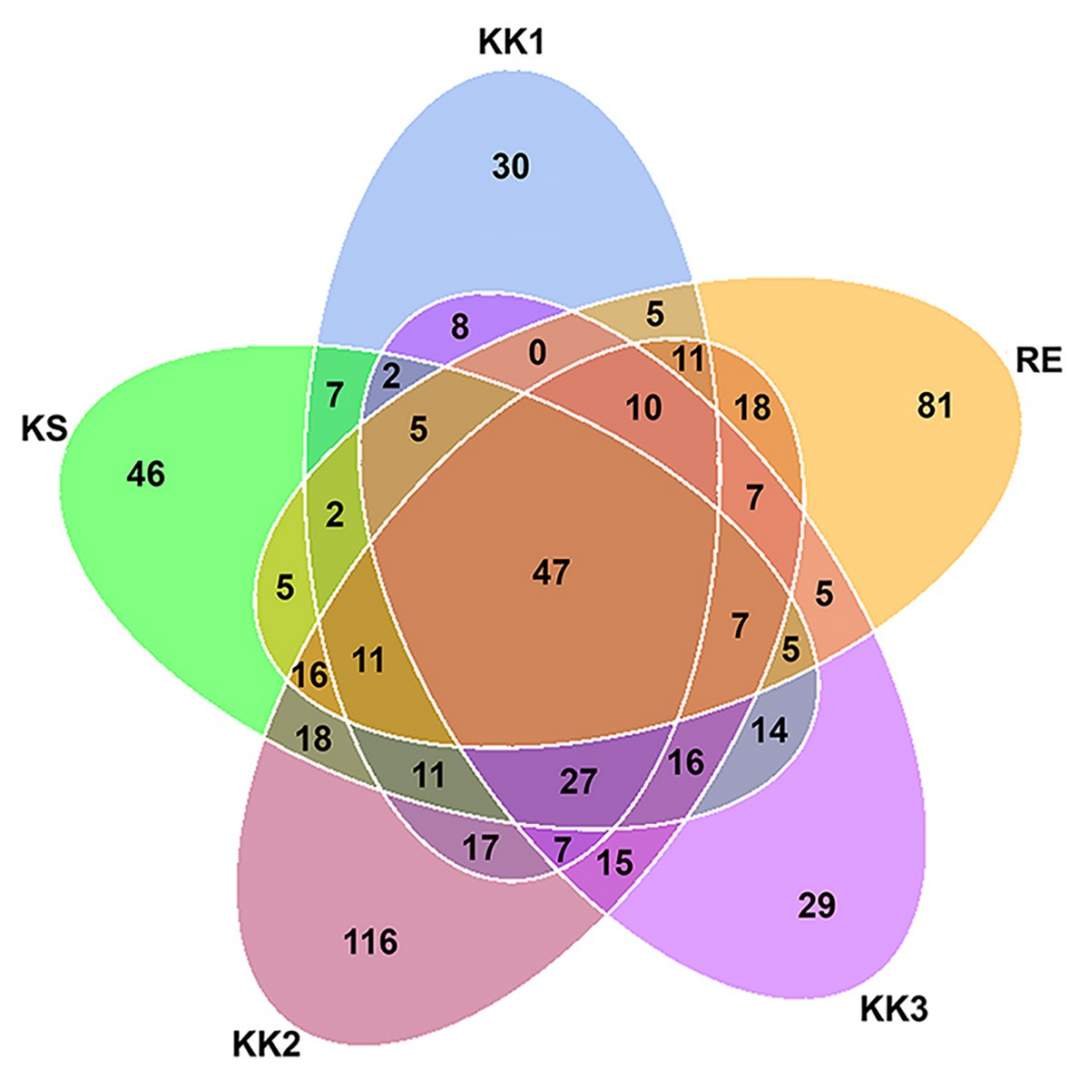
Venn diagram of distribution of bacterial OTUs among *pla-ra* samples from 5 different areas.

Forty-seven OTUs were present in all samples (Fig 2 and S2 Table). Each sample also contained some unique OTUs (Fig 2). The highest number of OTUs was detected in sample KK2 (354 OTUs, 116 being unique) (Table 2 and Fig 2). In contrast, the other samples had fewer unique OTUs—30, 29, 81 and 46 OTUs for the samples KK1, KK3, RE and KS, respectively. The unique OTUs are listed for each sample in S3 Table.

**Fig 3:**
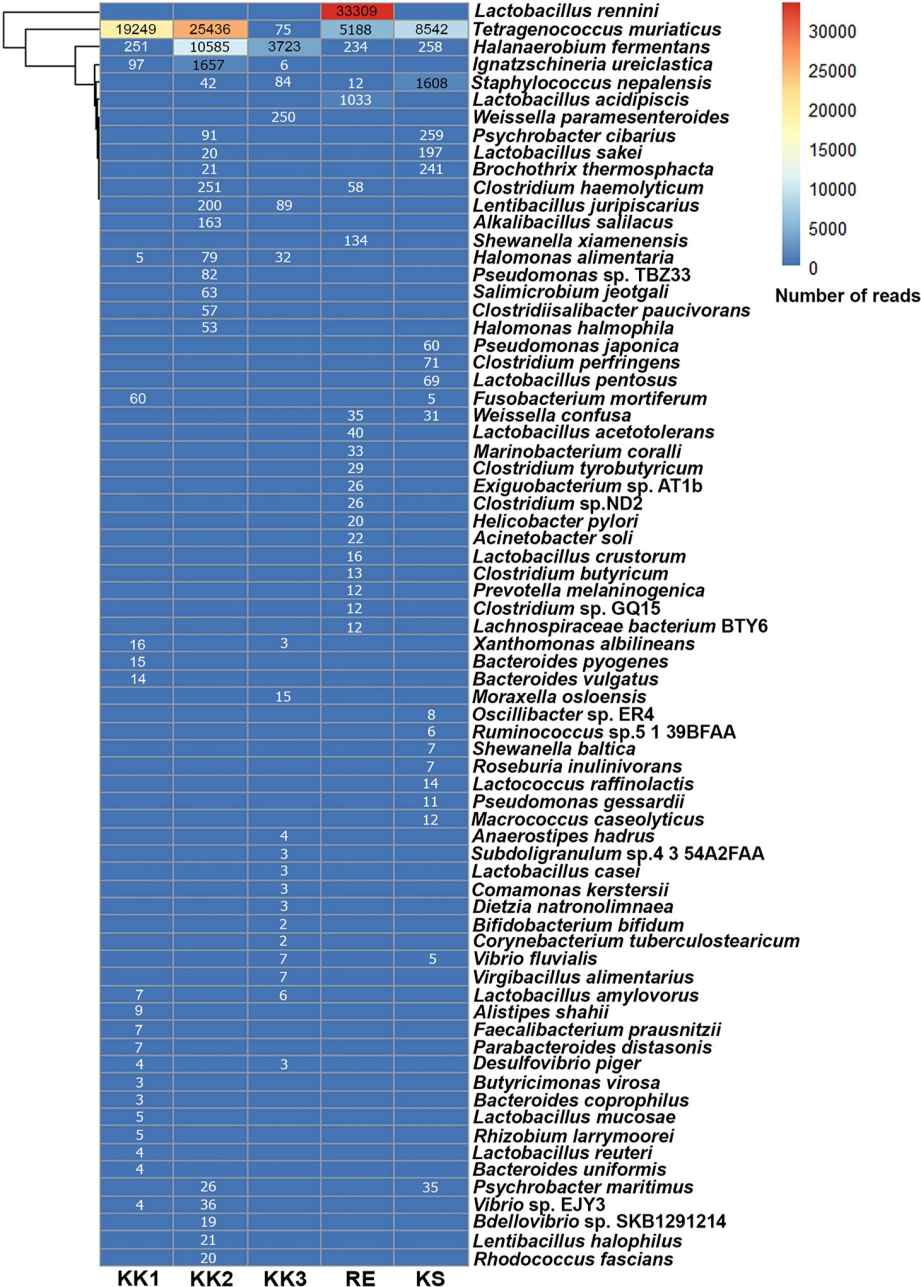
Heatmap of top 20 non-redundant species of bacteria from the five *pla-ra* samples, ranked by the number of reads within each sample. Numbers in the heatmap are reads per sample.

All OTUs at each taxonomic rank were annotated using comparisons against the SILVA database. The top 20 non-redundant taxa are specified in Fig 3. As illustrated in Figs 1 and 3, each *pla-ra* sample appeared to contain two to three dominant bacterial species. In particular, *Tetragenococcus muriaticus*, *Halanaerobium fermentans* and *Lactobacillus rennini* were prominent in one or more samples. The *pla-ra* samples KK1, KK2 and KS had *T*. *muriaticus* as the dominant species, whereas KK3 had *H*. *fermentans* and RE had *L*. *rennini* as the dominant species.

## Discussion

The present study used the IonS5^TM^XL sequencing platform for a comprehensive study of the bacterial community in *pla-ra* from five different areas across three provinces of northeastern Thailand. The pH, salinity and L-/D-lactic acid content of each *pla-ra* sample were measured. Nevertheless, these varied between samples: there are no standardized contents and recipes for *pla-ra* products [1, 5]. The pH level was likely affected by the lactic acid concentration: sample KK3 had the highest pH (7.15) but the lowest lactic acid content (90 mg/l) and salt concentration (7%). The starting salt concentration may one of the factors that leads to varying levels of lactic acid bacteria growth and their products, which in turn affects other sensory parameters in *pla-ra* [3, 7].

Seventeen phyla of bacteria as well as many genera and species were found in the samples. Community composition varied between samples, and also in comparison with previous reports [1, 5]. However, twenty-two genera from 47 OTUs were present in all our *pla-ra* samples (S2 Table) and among these were both beneficial (such as *Lactobacillus*) and harmful species (such as *Clostridium* and *Escherichia-Shigella*) [20, 21]. The phylum Firmicutes dominated the bacterial community, and especially the genera *Tetragenococcus*, *Halanaerobium*, *Lactobacillus*. These three genera, as well as *Weissella*, *Pediococcus*, *Enterococcus*, *Aerococcus*, *Clostridium*, and *Sphingomonas*, have been previously isolated from fermented fish (*pla-ra*, *pa-daek*, *pla-som*, *pla-chom*). Lactic-acid bacteria such as *Lactobacillus* and *Tetragenococcus*, producing lactic acid as a by-product of their carbohydrate metabolism, are the main genera in fermented food [1, 22–24]. We found high levels of D-/L-lactic acid in the *pla-ra* samples (KK1, KK2, RE and KS) in which *Tetragenococcus* and *Lactobacillus* were dominant, whereas sample KK3 was dominated by *Halanaerobium*, a halophilic taxon, and contained a lower concentration of lactic acid. This result may be due to different compositions of starting materials and recipes for *pla-ra* [1, 3, 7].

*Tetragenococcus muriaticus* is a halophilic lactic-acid species found in various salted and fermented fish [10, 25, 26]. It was detected in all samples and was the principal species in *pla-ra* samples KK1 and KS. These samples contained 9% salt and 450 mg/l D-/L-lactic acid but differed in their pH (5.13 and 4.83, respectively). However, the optimal growth parameters of this species appear to be broad. According to a previous report investigating *pla-ra* from central Thailand and Lao PDR, *T*. *muriaticus* is viable in salinities ranging from 11.3 to 20.3%, D-/L-lactic acid concentrations 0.40–0.88 g/100 g (equivalent to 4000 mg/l—8800 mg/l) and pH values between 5.6 and 6.1 [1]. Satomi et al. [27] reported an optimal pH between 7.5 and 8.0 and salt concentration about 7 to 10%: but *T*. *muriaticus* was able to grow in a wide range of pH (4.2 to 8.5) and in 1% to 25% salt while producing L-lactic acid. Furthermore, as the fermentation period increases to 12 months or longer, *Tetragenococcus* can rapidly grow and replace other taxa to become the most abundant genus [10]. This genus also reduces harmful substances such as ammonia and amines in fish sauce: numbers of *Tetragenococcus* and trimethylamine levels during fish sauce fermentation are negatively correlated [10]. *Tetragenococcus* may be a good bacterium to be used in *pla-ra* production [10].

*Halanaerobium fermentans* was the dominant species in *pla-ra* sample KK3, and was co-dominant with *T*. *muriaticus* in *pla-ra* sample KK2. *Halanaerobium fermentans*, a strictly anaerobic and moderately halophilic bacteria, can be isolated from salted puffer fish ovaries and its optimum growth condition is at 10% (w/v) (7–25%) salt, 35°C (15–45°C) and pH 7.5 (6.0–9.0) [28]. Unsurprisingly, the *pla-ra* from KK3 had the highest pH and lowest D-/L-lactic acid level, making it more suitable for *H*. *fermentans* growth. Sample KK2 contained as a co-dominant species *T*. *muriaticus*. This sample had 10% salt, 337.5 mg/l D-/L-lactic acid, and pH 5.02. *Halanaerobium* appears to be the dominant taxon until the 12th month of fish sauce fermentation, after which it is replaced by other taxa (such as *Tetragenococcus*) [10]. Similarly, in another fermented seafood, traditional Korean salted shrimp, that is normally fermented for 4–5 months, *Halanaerobium* is one of the dominant taxa after 49 days of fermentation [29]. Jung et al. [29] suggested that the *Halanaerobium* may be responsible for spoilage during fermentation. The presence of *Halanaerobium* and its products could be an indicator of over-fermentation of seafood.

Interestingly, the *pla-ra* microbiome in the sample from RE consisted mostly of *Lactobacillus rennini*, and other *Lactobacillus* species such as *L*. *acidipiscis*, *L*. *acetotolerans* and *L*. *crustorum*. The first of these was also detected in *pla-ra* products from Thailand and Lao PDR using polymerase chain reaction-denaturing gradient gel electrophoresis [1]. The genus *Lactobacillus* occurs naturally in various food products. Members of *Lactobacillus* are considered to be harmless and providing health benefits to humans and animals [20]. Indeed, they are commonly used as probiotics in food [30]. The dominant species, *L*. *rennini*, was originally isolated from rennet and associated with cheese spoilage [31]. It is a homofermentative species that can produce both D- and L-lactic acid. It is also related to *L*. *casei* and *L*. *plantarum*, which are probiotics and extensively used in the food industry as a microbial starter. *Lactobacillus rennini* possesses higher resistance to salt than do the other species [31–33]. Its presence as a dominant species in the *pla-ra* product from RE may benefit human health and could lead to improvements in fermented fish industrial applications. *Lactobacillus acidipiscis* was originally found in fermented fish (*pla-ra* and *pla-chom*) in Thailand and is capable of producing L-lactic acid from glucose [34]. The *pla-ra* product from RE contained more L-lactic acid (225 mg/l) than D-lactic acid (112.5 mg/l), possibly produced by *L*. *acidipiscis* but also by other species that cannot be specified.

*Staphylococcus nepalensis* was commonly found in all *pla-ra* samples, although with low read counts in each case. The genus *Staphylococcus* is naturally found in the environment and also in fermented fish, fermented seafood and fish sauce [35–38]. *Staphylococcus* species (*S*. *carnosus* and *S*. *xylosus*) are often used as starter cultures for enhancing the flavor and color of sausage products, controlling the fermentation process and improving product safety [39, 40]. Moreover, there is some evidence that *Staphylococcus* (*S*. *nepalensis* and *S*. *xylosus*) are able to improve odor by affecting volatile compounds in the fish sauce [35, 36]. Their presence in our *pla-ra* samples might be related to odor and taste that is different in each region of Thailand.

*Ignatzschineria ureiclastica* is another species found in all *pla-ra* samples. This genus is commonly isolated from flesh flies [41]. It may have been acquired from the environment.

Other species of interest include *Weissella paramesenteroides*, *Weissella confusa*, *Brochothrix thermosphacta*, *Lactobacillus sakei*, *Lactobacillus pentosus*, *Lentibacillus juripiscarius*, *Psychrobacter cibarius* and *Bifidobacterium bifidum*. These are all lactic-acid and halophilic bacteria common in fermented foods. Some have been used as probiotics for humans and animals [34, 42–47]. Additionally, to our knowledge, many genera we found in this study have not been previously reported in *pla-ra*; these include *Fusobacterium*, *Subdoligranulum*, *Ruminococcaceae_*UCG-014, *Erysipelotrichaceae_*UCG-003 and *Bifidobacterium* (Fig 1 and S1 Table).

Pathogenic bacteria were identified in *pla-ra* such as *Clostridium haemolyticum*, *Clostridium perfringens*, and *Vibrio fluvialis* [21, 47–49]. These contaminants may cause foodborne disease when using *pla-ra* as an ingredient for uncooked food. In particular, the acceptable microbiological concentration of *C*. *perfringens* is not more than 100 cfu/g *pla-ra* according to food hygiene criteria produced by Thai agricultural standard committees [6]. However, there is no history of severe illness caused by *pla-ra* in Thai communities. It seems that the preparation process and recipe destroy and prevent the growth of pathogens [7, 50]. Moreover, infection by these pathogenic agents may result in no symptoms, or mild to moderate symptoms, depending on the pathogenic potential of organisms, the host’s immune system, and the microbiota of local people [51–53]. Next-generation sequencing technology could potentially be used for screening for food pathogens in the *pla-ra* and/or other fermented fish products as a strategy to improve food safety standards.

Many species (598 OTUs) were found in the five *pla-ra* samples and there was considerable diversity in the microbiota among these samples. This may be due to different raw materials used, salt concentrations, recipes, processes and fermentation periods. The knowledge of microbial communities in *pla-ra* samples provides better understanding of these traditional products. However, the presence of some unclassified OTUs were found in present study and these microorganisms involved initiate fermentation are still unknown. The experiment was done without replicates, variation in diversity was because of different raw materials used for fermentation. The lacking data and biological replicates need to be clarified in the further investigations. Optimization of the fermentation process, using some dominant bacterial taxa in starter cultures, may help improve processes of food fermentation, food quality and flavor control, providing beneficial outcomes for industrial applications.

## Supporting information

S1 Table Complete list of bacterial OTUs found across all *pla-ra* samples. (XLSX)

S2 Table List of OTUs shared by all *pla-ra* samples. (XLSX)

S3 Table List of unique OTUs in each *pla-ra* sample. (XLSX)
